# Targeting Viral Antigens to CD11c on Dendritic Cells Induces Retrovirus-Specific T Cell Responses

**DOI:** 10.1371/journal.pone.0045102

**Published:** 2012-09-17

**Authors:** Asim Ejaz, Christoph G. Ammann, Roland Werner, Georg Huber, Verena Oberhauser, Susanne Hörl, Simone Schimmer, Ulf Dittmer, Dorothee von Laer, Heribert Stoiber, Zoltán Bánki

**Affiliations:** 1 Division of Virology, Innsbruck Medical University, Innsbruck, Austria; 2 Department of Internal Medicine I, Innsbruck Medical University, Innsbruck, Austria; 3 Institute of Virology, University of Duisburg-Essen, Essen, Germany; Imperial College London, United Kingdom

## Abstract

Dendritic cells (DC) represent the most potent antigen presenting cells and induce efficient cytotoxic T lymphocyte (CTL) responses against viral infections. Targeting antigens (Ag) to receptors on DCs is a promising strategy to enhance antitumor and antiviral immune responses induced by DCs. Here, we investigated the potential of CD11c-specific single-chain fragments (scFv) fused to an immunodominant peptide of Friend retrovirus for induction of virus-specific T cell responses by DCs. *In vitro* CD11c-specific scFv selectively targeted viral antigens to DCs and thereby significantly improved the activation of virus-specific T cells. In vaccination experiments DCs loaded with viral Ag targeted to CD11c provided improved rejection of FV-derived tumors and efficiently primed virus-specific CTL responses after virus challenge. Since the induction of strong virus-specific T cell responses is critical in viral infections, CD11c targeted protein vaccines might provide means to enhance the cellular immune response to prophylactic or therapeutic levels.

## Introduction

Dendritic cells (DC) are the most potent antigen presenting (APC) cells and play a central role in the induction of specific immune responses [1], [2]. Expression of an array of surface receptors (R), like receptors for C-type lectins (mannose R, DC-SIGN, DEC-205), Toll-like receptors (TLR), receptors for the Fc portion of antibodies (FcR) and complement Rs (CR3 and CR4) allow DCs to efficiently bind antigens (Ag) [3], [4], [5], [6]. Captured Ags are subsequently processed and efficiently presented to T cells due to the effective co-stimulatory capacity of mature DCs. Therefore, targeting protein Ags to receptors on DCs has emerged as a potential vaccination tool to induce antitumor and antiviral immune responses. Receptors like DEC-205, langerin (CD207) and CRs among others are intensively studied for their capacity to modify and strengthen humoral as well as specific T cell responses [7], [8], [9], [10], [11].

Complement C3 has been discussed to be involved in the induction of cytotoxic T lymphocytes (CTL) in viral infections with lymphocytic choriomeningitis virus (LCMV) or influenza [12], [13]. Furthermore, recent evidence has shown that C3-fragments on the surface of retroviruses like HIV and Friend virus (FV) enhance infection of DCs most likely through CD11c and CD11b binding, which subsequently leads to an improved virus-specific CD8^+^ T cell activation by DCs [14].

CD11c is the α-chain of CR4 (CD11c/CD18). CR4, together with CR3 (CD11b/CD18) and LFA-1 (CD11a/CD18) belongs to the heterodimeric receptor family of β_2_-integrins [15]. Similarly to CR3, inactivated C3b fragments (iC3b) deposited on the surface of antigens represent the main binding partner for CD11c. CD11c further interacts with C3b, ICAM-1 (CD54) and ICAM–2, proteins of the clotting system like fibrinogen, kininogen and factor X and molecules of microbial origin. Due to the usual co-expression with CD11b, the absence of commercially available CR4-knockout models and the lack of CD11c neutralizing Abs, data concerning the immunological role of CR4 are very limited.

In mice, CD11c is preferentially expressed on myeloid DCs, including both CD8^+^ and CD8^-^ subpopulations and is often used as DC-specific marker [2]. CD11c is highly expressed on DCs, nevertheless at lower levels certain sub-populations of B cells, NK cells and T cells display this receptor on their surface [16], [17], [18]. In experimental tumor models, targeting antigens to CD11c by specific Abs conjugated to Ags, single-chain Ab fragments (scFv) recombinantly fused to Ags or liposomes incorporating Ags have been demonstrated to successfully induce specific immune responses [11], [19], [20]. Thus far targeting Ags to CD11c has merely been tested in tumor models. Here we investigated the potential of targeting viral proteins to CD11c on DCs to trigger virus-specific CTL responses using the Friend virus model.

Friend virus (FV) represents a mouse model for retroviral infections [21]. FV consists of two viruses: a non-pathogenic helper virus, so called Friend murine leukemia virus (F-MuLV) and the pathogenic, replication-defective spleen focus-forming virus (SFFV). Co-infection of adult mice with these two viruses leads to polyclonal proliferation of erythroid precursor cells causing splenomegaly. In susceptible mouse strains, disease develops into lethal erythroleukemia. Disease resistant strains can control acute infection, but due to the induction of regulatory T cells, which down-regulate virus-specific CTL activity, a chronic infection develops [22], [23]. This well described retroviral infection model has been proven to be suitable to study specific immune responses and to test novel vaccination strategies.

In this study, we generated CD11c-specific scFv (CD11c-scFv) fused to the immunodominant region (IDR) of FV gag containing a CD8 T cell epitope (IDRgag). Using DCs treated with CD11c-scFv-IDRgag we detected significantly improved activation of FV-specific CD8^+^ T cells both *in vitro* and *in vivo*. FV-specific cytotoxic T lymphocytes (CTL) activated by DCs treated with the CD11c-scFv-IDRgag construct showed efficient rejection of FV-derived tumor cells *in vivo.* Furthermore, mice vaccinated with DCs loaded with the CD11c-scFv-IDRgag construct efficiently primed virus-specific CTL response after virus challenge.

**Figure 1:**
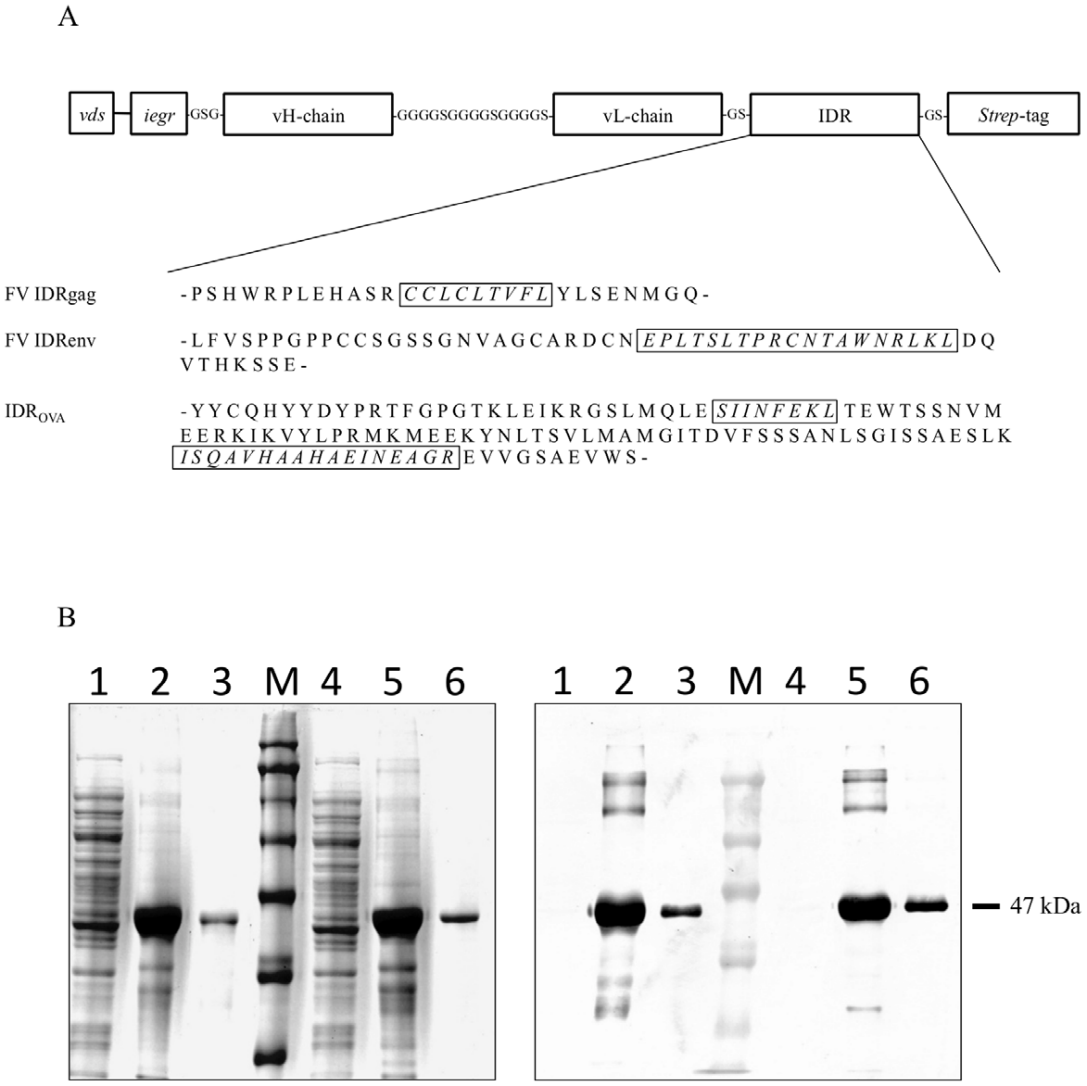
Generation and analyses of CD11c- and control-scFv-IDRgag fusion proteins. (**A**) Schematic representation of CD11c and control-scFv constructs fused to IDRs of either FV or OVA containing immunodominant epitopes. Abbreviations: vds (vector derived sequence); iegr (factor Xa cleavage site); IDR (immunodominant region). (**B**) The recombinant CD11c- and control-scFv-IDRgag (lanes 4, 5, 6 and 1, 2, 3, respectively) were expressed in the bacteria as inclusion bodies, which were isolated and dissolved in 8 M urea. The proteins were purified using a HisTrap HP column. Purified recombinant proteins were then refolded by slow dialyses. The verification of the purified proteins was performed using SDS-PAGE (left) followed by Western blotting (right). (Lanes 1 and 4 bacterial lysate; lanes 2 and 5 isolated inclusion bodies dissolved in 8M urea; lanes 3 and 6 purified, refolded proteins; M molecular weight marker).

## Methods

### Mice

Experiments were conducted using 3- to 6-month-old female C57BL/6 (B6) mice (Harlan Laboratories, Italy), FV-specific T cell receptor (TCR) transgenic (tg) CD8 mice [24], carrying a TCR transgene encoding for a TCR that recognizes the gag leader peptide of FV [25]. FV-specific CD4^+^ TCRβ-tg mice carrying a TCR transgene specific for the Env protein of F-MuLV [26] were also used. Furthermore OVA-specific CD8 TCRtg OT-1 and CD4 TCRtg OT-2 animals were included.

**Figure 2:**
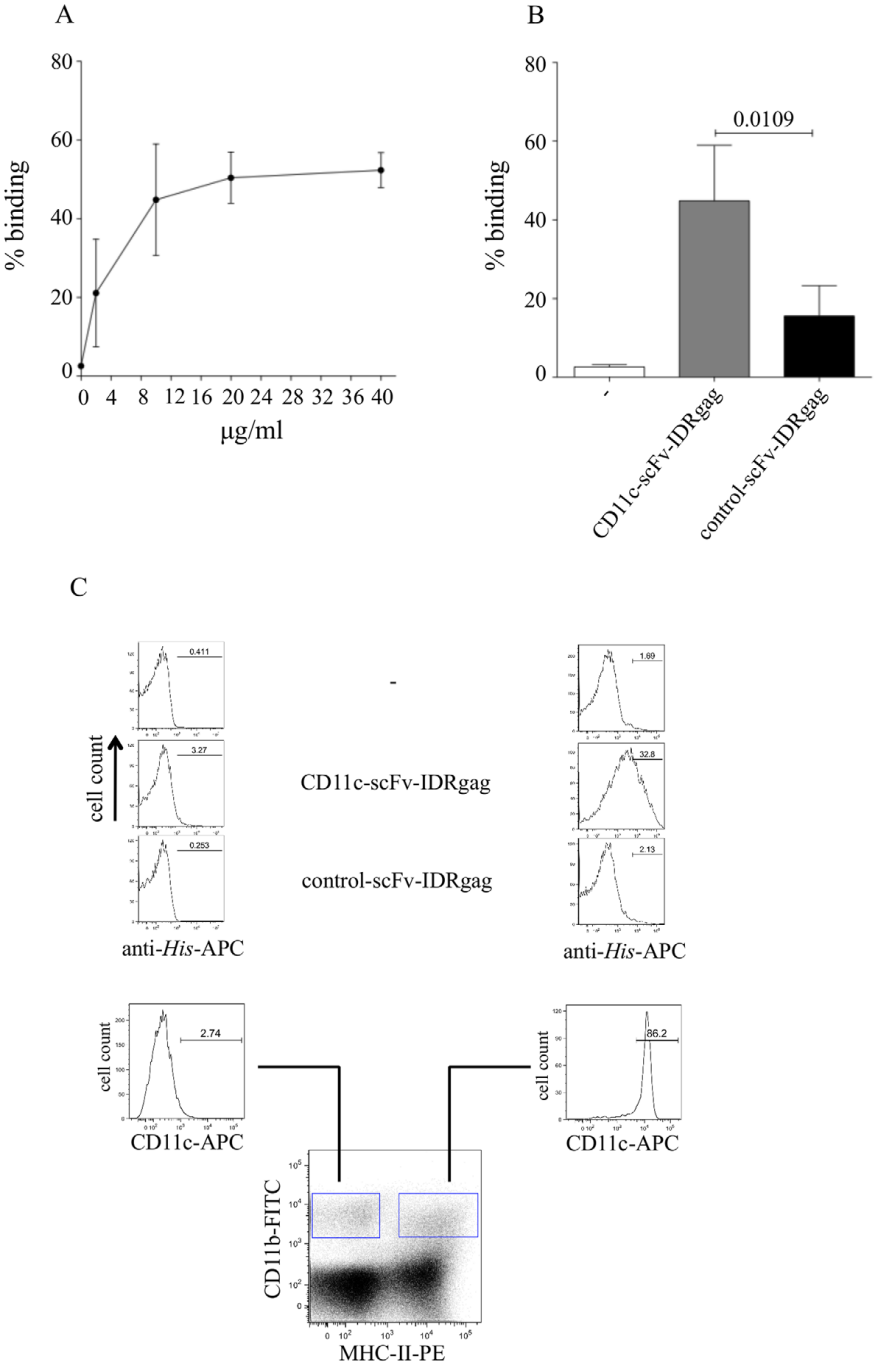
Binding of CD11c-scFv-IDRgag to bmDCs and spleen DCs. (**A**) Binding of CD11c-scFv-IDRgag to bmDCs at different concentrations. Data represent mean±SD values of 4 independent experiments. (**B**) At 10 µg/ml concentration, CD11c-scFv-IDRgag bound to bmDCs with significantly higher capacity as compared to the control-scFv-IDRgag. Data represent mean±SD values of 4 independent experiments. Data were analyzed with unpaired *t*-test. (**C**) Using spleen cell suspensions, we investigated binding-specificity of CD11c-scFv-IDRgag on DCs. By gating on CD11b and MHC class II we defined a population of CD11c expressing DC without staining for CD11c itself. Whereas CD11b^+^/MHC class II^-^ cells did not express CD11c, the CD11b/MHC class II double-positive population represented CD11c expressing DCs. We found that CD11c-scFv-IDRgag selectively targets CD11c positive cells *in vitro*. Results of one representative experiment are shown.

### Ethics Statement

All mice were bred and maintained free of specific pathogens in the animal facility at the Division of Virology, Innsbruck, Austria. Mice were treated in accordance with the guidelines of the “European Convention for the Protection of Vertebrate Animals used for Experimental and other Scientific Purposes” and the Austrian law. Animal experiments were approved by the ethics committee of the Austrian Federal Ministry of Science and Research (BMWF-66.0011/0152-II/3b/2010; BMWF-66.011/0042-II/3b/2011; BMWF-66.011/0076-II/3b/2011).

### Antibodies, Cell Lines and Reagents

Monoclonal antibodies against mouse CD4, CD8, CD69, CD25, CD11c and CD11b were purchased from BD Pharmingen if not indicated otherwise. Hamster anti-CD11c clone N418 hybridoma [27], *Mus dunni* cells [28] and FBL-3 cells [25] were cultured in RPMI 1640 supplemented with 10% FCS and 2 mM L-glutamine.

### Virus Stocks and C’-opsonization

F-MuLV stocks for *in vitro* opsonization were generated in permissive *Mus dunni* cells. Virus containing cell-culture supernatants (SNs) were stored at −80°C until use. F-MuLV was opsonized in the presence of normal mouse serum (NMS) (as source of complement) at a dilution of 1∶10 for 60 minutes at 37°C (F-MuLV-C). To remove NMS, virus was ultracentrifuged (23000×g, 2 hrs, 4°C) and the virus pellet was resuspended in medium. To confirm the opsonization pattern of the viruses, F-MuLV-C, and non-opsonized F-MuLV were applied in a virus capture assay (VCA) [14]. Real-time RT-PCR using FV-specific primers and fluorescently labelled Taq-Man probes were used to determine the amount of captured F-MuLV [14].

**Figure 3:**
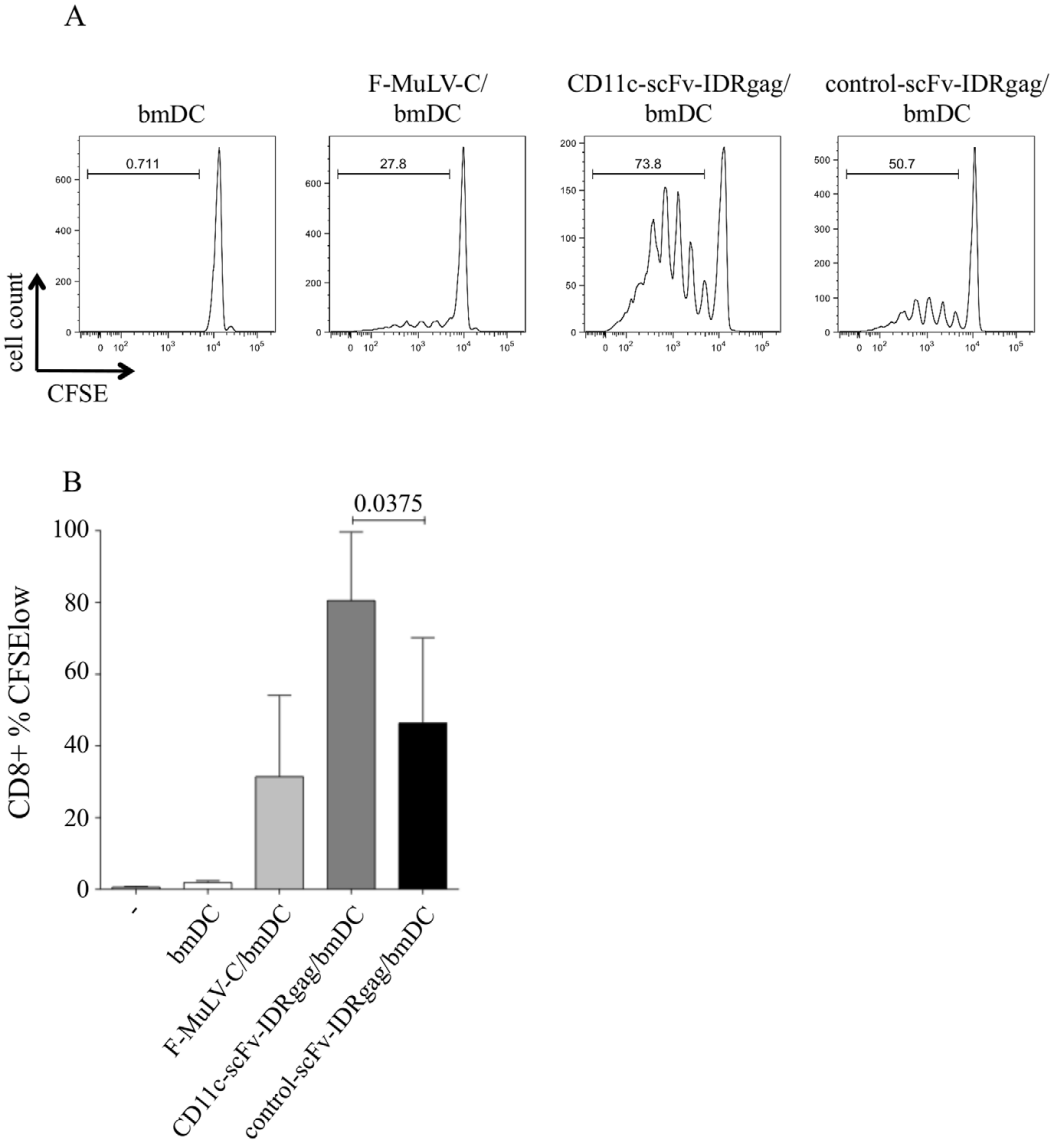
DCs loaded with CD11c-scFv-IDRgag induced proliferation of FV-specific CD8^+^ T cells. (**A**) To study proliferation of FV-specific CD8^+^ T cells induced by CD11c-scFv-IDRgag treated bmDCs, isolated FV-specific TCRtg CD8^+^ T cells were stained with CFSE prior to co-culture with bmDCs. After 4 days of co-culture both control-scFv-IDRgag and CD11c-scFv-gagID-loaded bmDCs induced proliferation of FV-specific CD8^+^ T cells. (**B**) The proliferation of CD8^+^ T cells induced by CD11c-scFv-IDRgag was significantly higher compared to DCs with control-scFv-IDRgag. Data represent mean±SD of 5 independent experiments. Data were analyzed with unpaired *t*-test.

### Preparation of Anti-CD11c Single-chain Fragment Variable (scFv)-IDRgag

The gene comprising the variable heavy- and light-chain of hamster anti-mouse CD11c mAb (cloneN418) joined by a linker (-GGGGSGGGGSGGGGS-), including the immunodominant region (IDR) of FV gag containing a immunodominant gag CTL epitope gPr80^gag^85–93 (CCLCLTVFL) [25] and a *Strep*-tag was manufactured by Geneart (Fig. 1A). The gene was cloned into the pET32a(+) (Novagen) expression vector using the BglII and NotI restriction sites. The plasmid was transformed and expressed in BL21(DE3) (Novagen). The same scheme was followed for the construction and expression of the control scFv-IDRgag using the sequence of variable heavy- and light-chain of anti-human β-gal mAb. To study FV-specific CD4^+^ T cell activation scFv constructs were generated including the IDR of FV env containing a known CD4 TCR epitope (EPLTSLTPRCNTAWNRLKL, referred as IDRenv) (Fig. 1A). Alternatively, IDRs containing both major CD8 and CD4 TCR epitopes (SIINFEKL and ISQAVHAAHAEINEAGR, respectively) referred to as IDR_OVA_ were also cloned into scFv constructs (Fig. 1A). FV matrix protein p15 (MA) [29] extended with the immunodominant region (IDR) of FV gag containing a immunodominant gag CTL epitope CCLCLTVFL (IDRgag-MAp15) was produced in BL21(DE3) using pET32a(+) expression cassette. We used this IDRgag-MAp15 as non-targeted control since the molecular weight of IDRgag-MAp15 (∼46.5 kDa) is close to CD11c-scFv-IDRgag (∼47 kDa), which makes putative non-specific uptake of the proteins by DCs (e.g. with non-targeted macropinocytosis) more comparable. The recombinant proteins were expressed in the bacteria as inclusion bodies, which were isolated, washed twice with 2 M urea and dissolved in 8 M urea. The proteins were purified using a HisTrap HP column (GE). Purified recombinant proteins were then refolded by slow dialysis (8M urea to 0M urea) in the presence of 0.1% Tween 80 (Sigma) at all steps. The verification of the purified proteins was performed using SDS-PAGE followed by Western blotting. The blots were stained with peroxidase-labeled anti-*Strep*-tag II mAb (IBA) and developed chromogenically using Diaminobenzidine (Sigma) as a substrate.

**Figure 4:**
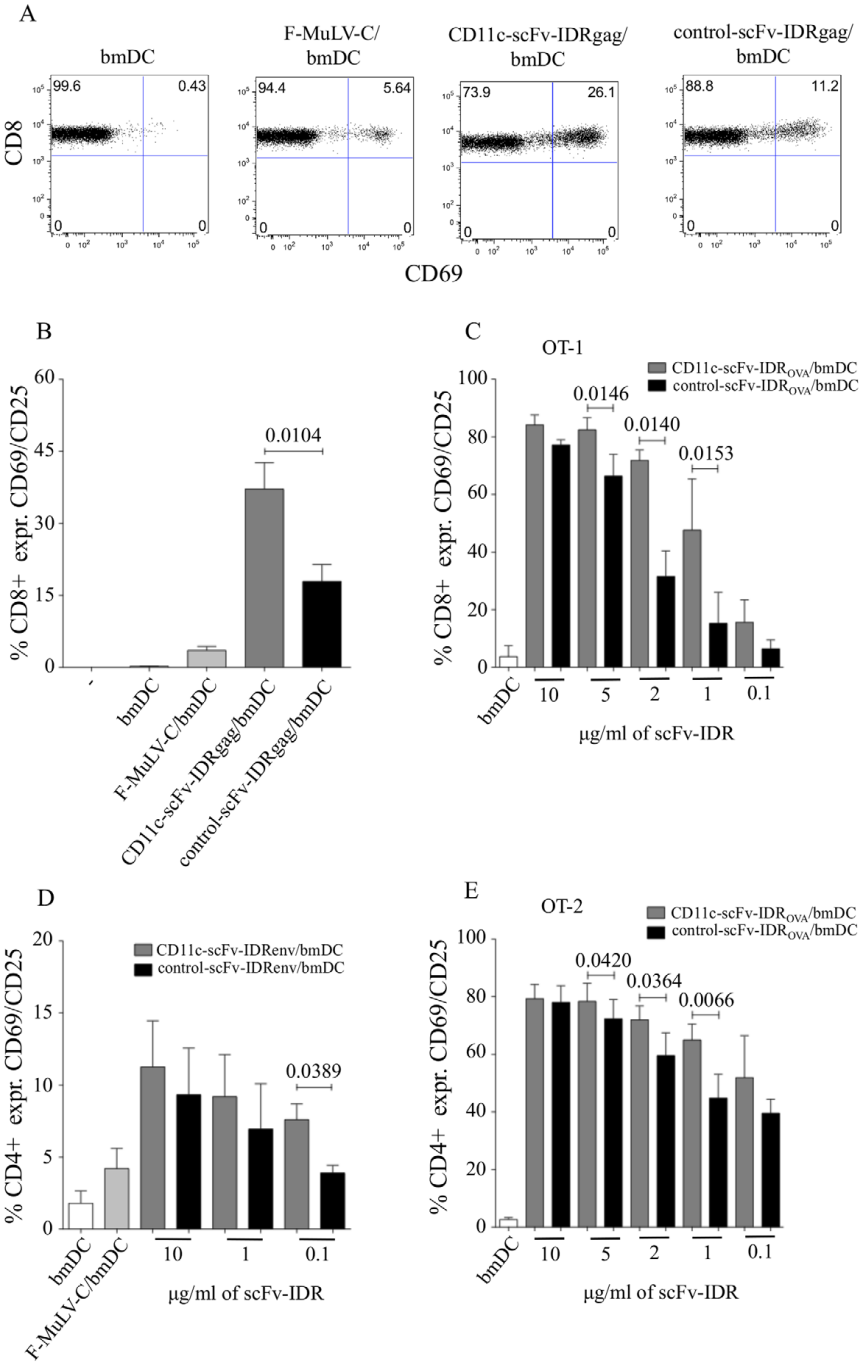
Targeting antigens to CD11c on DCs improved activation of specific T cells measured by up-regulation of CD69 and CD25 *in vitro*. (**A**) Activation of FV-specific TCRtg CD8^+^ T cells upon co-culture with DCs loaded either with C-opsonized F-MuLV (F-MuLV-C), 10 µg/ml CD11c-scFv-IDRgag or 10 µg/ml control-scFv-IDRgag. Data display one representative experiment showing the percentage of CD69 expressing CD8^+^ T cells. Co-expression of CD69 and CD25 either on FV-specific CD8^+^ T cells upon co-culture with DCs loaded either with C-opsonized F-MuLV (F-MuLV-C), 10 µg/ml CD11c-scFv-IDRgag or 10 µg/ml control-scFv-IDRgag (**B**). Co-expression of CD69 and CD25 either on FV-specific CD4^+^ T cells after 24 hours co-culture with differentially loaded DCs (**D**). Data represent mean±SD of 8 and 3 independent experiments for CD8^+^ T cell and CD4^+^ T cell co-cultures, respectively. Data were analyzed with unpaired *t*-test. Co-expression of CD69 and CD25 on OVA-specific CD8^+^ OT-1 (**C**) or CD4^+^ OT-2 (**E**) T cells after 24 hours co-culture with differentially loaded DCs. Data represent mean±SD of 3 and 4 independent experiments for CD8^+^ T cell and CD4^+^ T cell co-cultures, respectively. Data were analyzed with unpaired *t*-test.

### Generation of Mouse Bone Marrow-derived DCs and Isolation of Mouse CD8^+^ and CD4^+^ T Cells

Bone-marrow derived DCs (bmDC) were generated as described by Inaba et al. with some modifications [30]. Briefly, 2×10^6^ bone marrow cells were cultivated in 10 ml of DC medium (RPMI 1640 supplemented with 10% FCS, 2 mM L-glutamine, 500 nM 2-ME, 100 U/mL penicillin/streptomycin and 1000 U/ml recombinant mouse GM-CSF and recombinant mouse IL-4) in Petri dishes for three days at 37°C. 10 ml of fresh DC medium was then added and cells were cultured for another 3 days. On day 6 non-adherent cells were removed, washed and cultivated in fresh 20 ml DC medium. Non-adherent cells obtained after 8 days displayed a myeloid DC phenotype (>85% CD11c and >95% CD11b) as revealed by flow cytometry.

FV-specific CD8^+^ and CD4^+^ T cells were isolated from the spleens of FV-specific CD8^+^ and CD4^+^ TCR tg mice using the BD IMag CD8 and CD4 T Lymphocyte Enrichment Set (BD Pharmingen) according to the manufacturer’s instructions (purity >95% as determined by FACS). OVA-specific CD8^+^ and CD4^+^ T cells were isolated from the spleens of OVA-specific TCR tg OT-1 and OT-2 mice.

**Figure 5:**
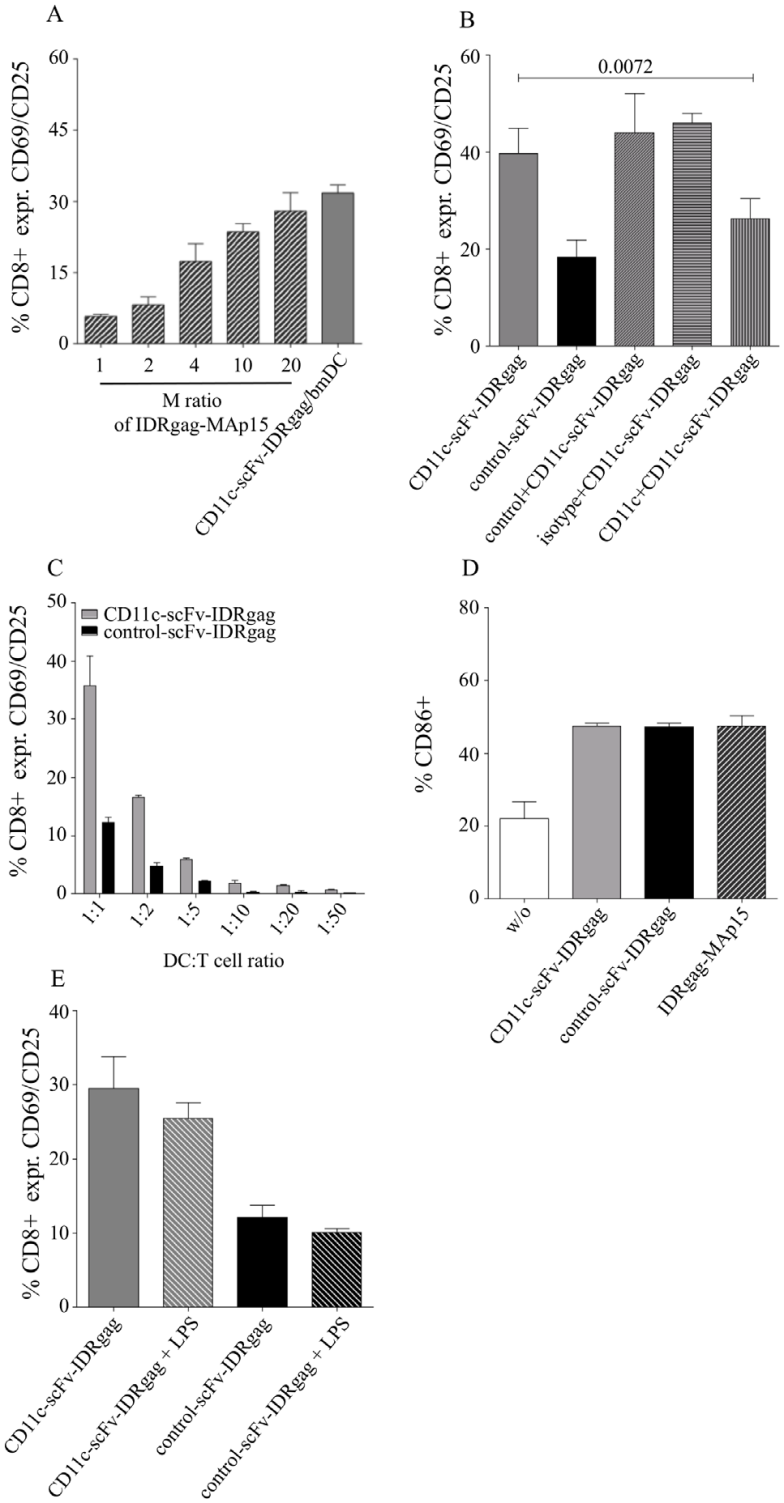
Analysis of FV-specific CD8^+^ T cell activation induced by targeting viral Ag to CD11c on DC. (**A**) Activation induced by DCs loaded with 10 µg/ml of CD11c-scFv-IDRgag was equivalent with DCs loaded with 20 times molar excess of non-targeted FV matrix protein p15 (MA) extended with the same immunodominant CCLCLTVFL FVgag epitope (IDRgag-Map15). Data represent mean±SD of 2 independent experiments. (**B**) We tested the receptor-specificity of IDRgag targeting by incubating DCs with CD11c-scFv-IDRgag either in the presence of parental anti-CD11c mAb clone N418 or control mAb (anti-human β-gal mAb) as well as isotype control Ab (Armenian hamster IgG). Whereas anti-CD11c parental mAb was able to significantly reduce CD11c-scFv-IDRgag induced CD8^+^ activation in co-culture experiments, no decrease of CD8^+^ activation was observed using the parental control mAb or isotype control Ab. Data represent mean±SD of 5 independent experiments. Data were analyzed with unpaired *t*-test. (**C**) Maturation of DCs pulsed with either 10 µg/ml control- or CD11c-scFv-IDRgag as well as IDRgag-MAp15 was measured after 24 hours by determining the expression of co-stimulatory molecule CD86 on the cell-surface. Data represent mean±SD of 3 independent experiments. (**D**) Effect of the addition of 10 ng/ml LPS to DCs pulsed with either control- or CD11c-scFv-IDRgag on their capacity to activate FV-specific CD8^+^ T cells. Data represent mean±SD of 3 independent experiments.

### Binding Assay of scFv-IDRgags to bmDCs

To analyze the binding of scFv-IDRgags to bmDCs, 0.5×10^6^ bmDCs or 10^6^ mouse spleen cells were incubated with different concentrations of CD11c-scFv-IDRgag or control-scFv-IDRgag at 4°C for 30 min. The cells were then washed and incubated with biotinylated anti-His mAb (Miltenyi Biotech) for 30 min at 4°C. After washing cells were incubated with PE-labeled anti-mouse CD11b (cloneM1/70, BD Pharmingen), 7-AAD (BD Pharmingen) and APC-labeled Streptavidin (BD Pharmingen) for further 30 min at 4°C. Cells were then washed and analyzed by FACS.

### Co-culture of scFv-IDRgag Loaded bmDCs with FV-specific CD8^+^ and CD4^+^ TCRtg T Cells

For co-culture experiments, 0.5×10^6^ bmDCs in 500 µl of culture medium were loaded with 10 µg/ml of CD11c-scFv-IDRgag or control-scFv-IDRgag and incubated at 37°C for 24 h. The cells were washed twice and then co-cultured with 0.5×10^6 ^FV-specific TCRtg CD8^+^ T cells. After 24 h of co-culture, activation of FV-specific CD8^+^ T cells was analyzed by flow-cytometry. In some experiments bmDCs were pre-incubated with parental anti-CD11c mAb clone N418 or control mAb (anti-human β-Gal) for 30 min at 37°C and then loaded with CD11c scFv-IDRgag constructs. To study the induction of FV-specific CD4^+^ T cells bmDCs were incubated with CD11c-scFv-IDRenv or control-scFv-IDRenv. The same experimental settings were used to test CD11c-scFv-IDR_OVA_ and control-scFv-IDR_OVA_ constructs with OT-1 CD8^+^ and OT-2 CD4^+^ T cells.

**Figure 6:**
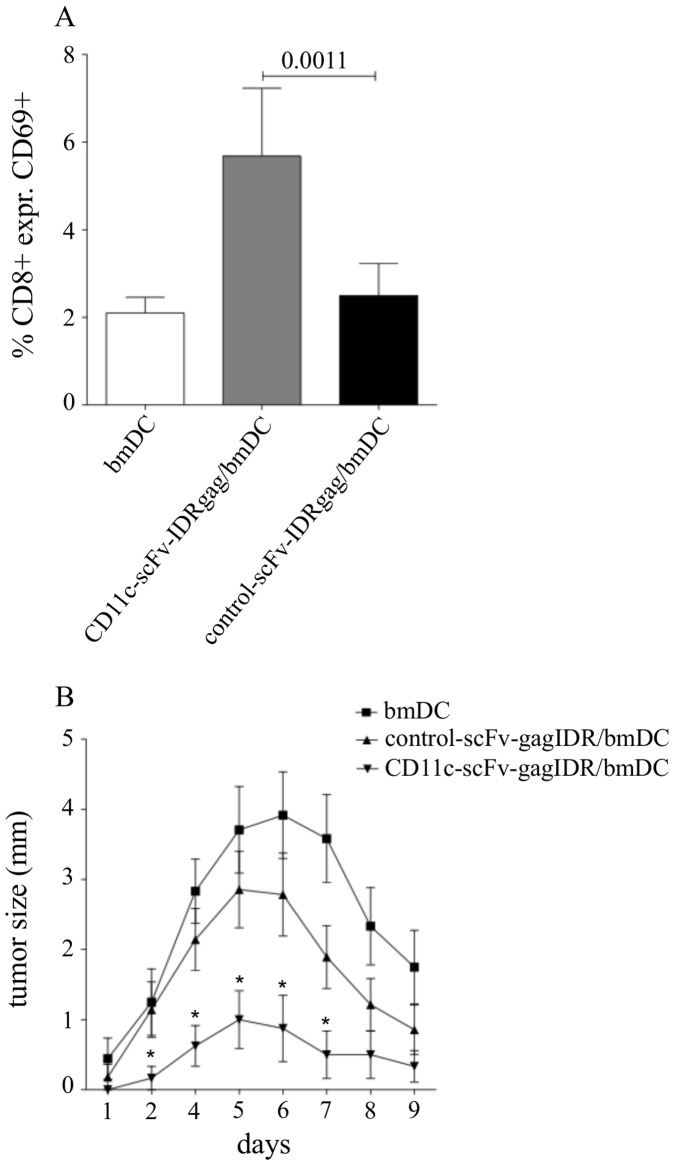
Improved rejection of FBL-3 tumor in B6 mice by CD11c-scFv-IDRgag loaded DCs. (**A**) CD8 TCRtg B6 mice injected with CD11c-scFv-IDRgag-treated bmDCs showed a significant higher level of CD8^+^ T cell activation, compared to mice receiving bmDCs loaded with control-scFv-IDRgag as measured by the percentage of CD69 expressing CD8^+^ T cells in the spleen. Data represent mean±SD values from 6 mice each group collected from 2 independent experiments. Data were analyzed with unpaired *t*-test. (**B**) B6 mice received bmDCs loaded either with CD11c-scFv-IDRgag or control-scFv-IDRgag. Control mice were injected with non-loaded bmDCs. 24 hours after receiving DCs mice were injected i.v. with FV-specific TCRtg CD8^+^ T cells. FBL-3 cells were inoculated i.d. 48 h after bmDCs injection. Tumor development was monitored daily for the next 9 days by measuring tumor size. Animals injected with DCs alone rejected the tumor within 11 days (data not shown) with a maximum tumor size of 3.81±0.61 mm at day 6. A significant difference in size of the tumors was observed at day 2, 4, 5, 6 and 7 (unpaired *t*-test) between the groups of animals receiving DCs loaded either with control-scFv-IDRgag or CD11c-scFV-IDRgag with a maximum tumor size of 2.85±0.54 mm or 1.00±0.41 mm at day 5, respectively. Data represent mean±SD values from 12 mice from each group collected from 3 independent experiments.

### CFSE-labeling

To study the proliferation, isolated CD8^+^ T cells were labeled with CFSE prior to co-culture and the dilution pattern of CFSE was analyzed by FACS after 4 days. Briefly, 10×10^6^ CD8^+^ T cells were stained with a final concentration of 2 µM CFSE in 4 ml PBS for 10 min at 37°C. Cells were washed twice with 15 ml RPMI/10%FCS. Unbound CFSE was removed by an incubation of cells with 15 ml RPMI/10% FCS for 5 min at 37°C followed by a wash with 15 ml RPMI/10% FCS. The cells were resuspended in RPMI/10% FCS and subjected to co-culture with loaded DCs.

### Adoptive Transfer and Tumor Challenge

Therapeutic potential of scFv-IDRgag loaded bmDCs was analyzed *in vivo* by investigating the rejection of tumors of the intradermally injected F-MuLV-induced erythroleukemia cell line, FBL-3 in B6 mice. Briefly, 0.5×10^6^ bmDCs were loaded for 24 hours either with CD11c-scFv-IDRgag, control-scFv-IDRgag *in vitro*. Cells were washed twice and injected intravenously into B6 mice. As additional control non-loaded bmDCs were applied in a parallel group of animals. The next day mice received 0.5×10^5^ FV-specific TCRtg CD8^+^ T cells intravenously. The hair on the back of mice was shaved with animal clippers and 48 hours post-DC transfer 1×10^7^ FBL-3 tumor cells were injected intradermally (i.d.). Tumor development was monitored daily for next 9 days by measuring tumor diameters at right angles with vernier-calipers to calculate the mean diameter.

### DC-vaccination and FV Challenge

B6 mice were vaccinated twice in one-week interval by i.v. injection of 0.5×10^6^ bmDCs loaded for 24 hours either with CD11c-scFv-IDRgag or control-scFv-IDRgag in the presence of 50 ng/ml LPS providing full DC maturation. As negative control 0.5×10^6^ LPS-activated, non-loaded bmDCs were used. One week after the second immunization mice were challenged with i.v. injection of 10.000 SFFU of FV-complex.

### Statistics

All statistical analyses were performed with GraphPad PRISM Software. Statistical analyses for differences between groups were performed using one-way ANOVA followed by Tukey’s post-test (3 experimental groups or more) or Student’s t-test (two experimental groups). Data are presented as mean+/−SD. Results with p<0.05 were considered to be statistically significant.

**Figure 7:**
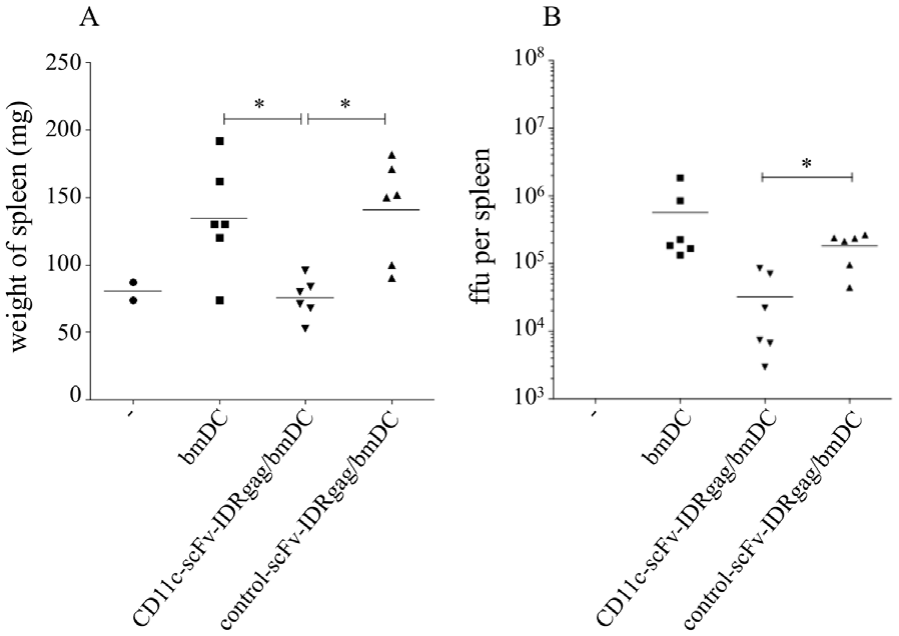
Immunization of mice with DCs loaded with CD11c-scFv-IDRgag provides a better control of acute viremia. (**A**) When animals were immunized twice with bmDCs loaded with CD11c-scFv-IDRgag or control-scFv-IDRgag in one week interval and challenged one week after the second immunization, animals immunized with bmDCs loaded with CD11c-scFv-IDRgag were protected significantly better against FV induced splenomegaly, which correlated with a significantly lower frequency of infected cells in the spleen (**B**). Data were analyzed by one-way ANOVA with Tukey’s multiple comparison test. Significance was labeled with * if p<0.05, ** if p<0.01, *** if p<0.001. Data represents results from one experiment.

**Figure 8:**
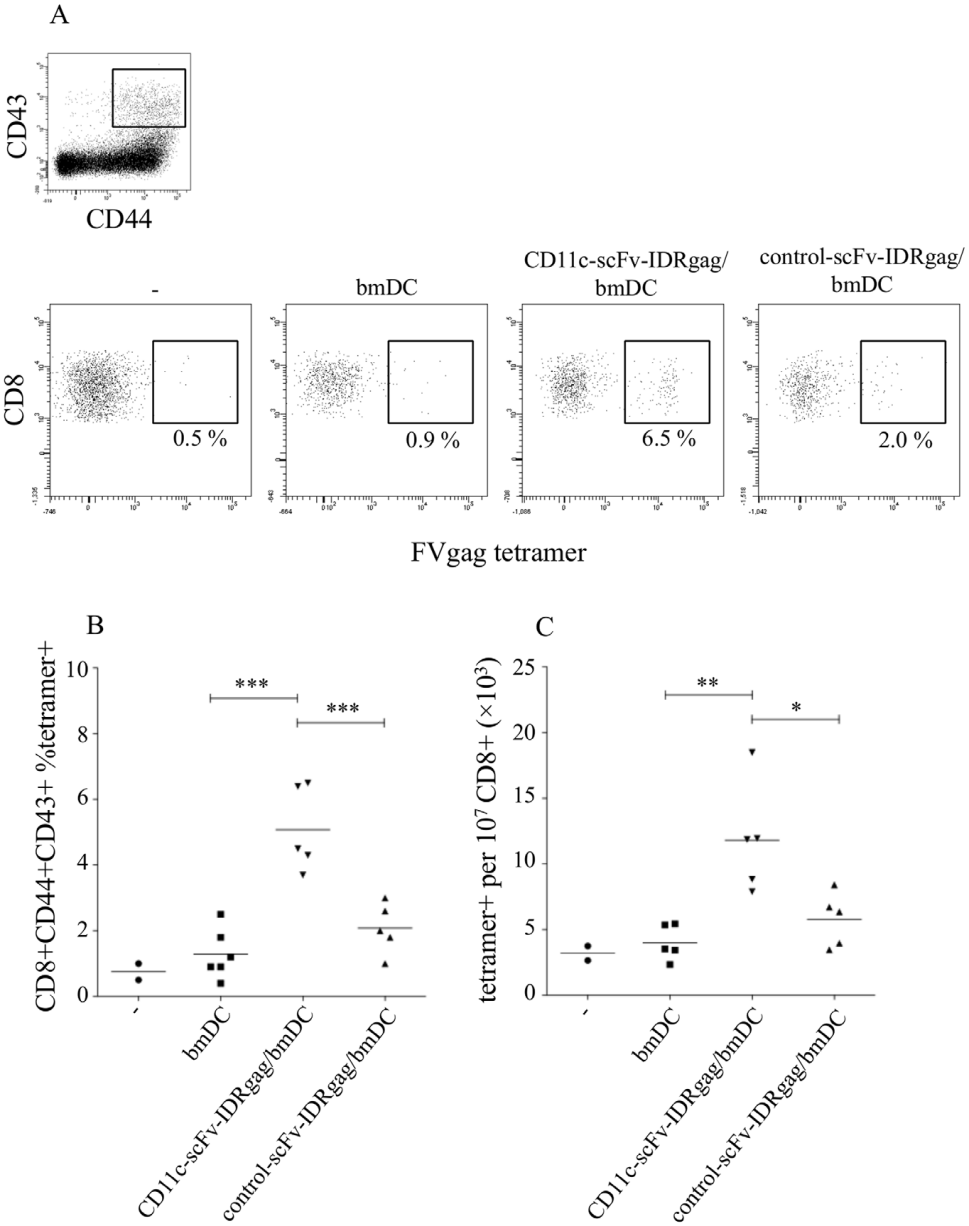
Improved induction of FV-specific CD8^+^ T cells by vaccination with CD11c-scFv-IDRgag treated DCs followed by FV challenge. (**A and B**) By gating on CD43/CD44 double positive effector CD8^+^ T cell population the frequency of FVgag tetramer^+^ cells was measured at 4 dpi. We detected significant induction of FV-specific CTL-response in mice injected with DCs loaded with CD11c-scFvIDRgag (5.08±0.57 percent specific FVgag tetramer^+^ CD8^+^ T cells), when compared to mice inoculated with control-scFv-IDRgag loaded DCs (2.08±0.43 percent specific FVgag tetramer^+^ CD8^+^ T cells). At 4 days post FV challenge mice injected with non-loaded DC showed nearly no detectable FV-specific CTL responses. Data were analyzed by one-way ANOVA with Tukey’s multiple comparison test. Significance was labeled with * if p<0.05, ** if p<0.01, *** if p<0.001. Data represents results from one experiment. (**C**) Investigating the total number of FV-specific CD8^+^ T cells per 10^7^ CD8^+^ T cells, we found significant higher number of FV-specific CTLs in mice vaccinated with bmDC/CD11c-scFv-IDRgag (11803±1855) compared to both mice inoculated non-loaded or control-scFv-IDRgag loaded DCs (4020±599 and 5782±916, respectively). Data were analyzed by one-way ANOVA with Tukey’s multiple comparison test. Significance was labeled with * if p<0.05, ** if p<0.01, *** if p<0.001. Data represents results from one experiment.

## Results

### Generation and Analysis of CD11c-scFv-IDRgag Fusion Proteins

CD11c-specific and control scFv fused to the IDRgag were produced in bacteria as inclusion bodies and purified using affinity chromatography. The purified recombinant proteins were analyzed by SDS-PAGE and showed the theoretically calculated molecular shift of 47 KDa (Fig. 1B left). The scFv was further analyzed by Western blot, which was developed using anti-*Strep*-tag mAb (Fig. 1B right). Next the binding capacity of CD11c-scFv-IDRgag to bone marrow-derived DCs (bmDC) was investigated. Serial dilution of CD11c-scFv-IDRgag revealed that a concentration of 10 µg/ml provided saturated binding to bmDCs, therefore this concentration was selected and used for subsequent experiments (Fig. 2A). At a concentration of 10µg/ml, CD11c-scFv-IDRgag showed significantly higher binding to bone marrow-derived DCs (bmDC) than the control-scFv-IDRgag (Fig. 2B). We also tested CD11c-scFv-IDRgag binding *ex vivo* to DCs in suspension of spleen cells isolated from naïve mice. By gating on CD11b and MHC class II we defined a population of CD11c expressing DC without staining for CD11c itself. Whereas CD11b^+^/MHC class II^-^ cells did not express CD11c, the CD11b/MHC class II double-positive population represented CD11c expressing DCs (Fig. 2C). Studying the binding of scFv-IDRgag to these cell populations, we found that CD11c-scFv-IDRgag selectively targeted CD11c positive cells (Fig. 2C).

### Induction of CD8^+^ T Cell Proliferation by CD11c-scFv-IDRgag Loaded DCs

Since a functional CD8^+^ T cell response was demonstrated to be essential for the control of acute FV infection [22], we first focused on the proliferation of FV-specific CD8^+^ T cells induced by CD11c-scFv-IDRgag treated DCs. To study proliferation of FV-specific CD8^+^ T cells induced by CD11c-scFv-IDRgag treated bmDCs, isolated FV-specific TCRtg CD8^+^ T cells were stained with CFSE prior to co-culture with bmDCs. We found that after 4 days of co-culture both control-scFv-IDRgag and CD11c-scFv-gagIDR-loaded bmDCs induced proliferation of FV-specific CD8^+^ T cells. However, the proliferation of CD8^+^ T cells induced by CD11c-scFv-IDRgag was significantly higher (Fig. 3A and 3B).

### Targeting IDRs of FV gag/env or OVA to CD11c on bmDC Induced Either FV-specific or OVA-specific T Cell Activation in vitro

We next examined the efficiency of targeting FV IDRgag to CD11c on DCs for its stimulatory capacity to activate virus-specific CD8^+^ T cells *in vitro*. For this, 0.5×10^6^ FV-specific TCRtg CD8^+^ T cells recognizing the immunodominant gag CTL epitope gPr80^gag^85–93 were co-cultured with 0.5×10^6^ bmDCs treated either with CD11c-scFv-IDRgag or control-scFv-IDRgag. After 24 hours of co-culture we measured the expression of the early activation marker CD69 on CD8^+^ T cells (Fig. 3A). Since we demonstrated previously that C-opsonized F-MuLV efficiently infects bmDCs and subsequently activates specific CTLs [14], bmDCs infected with 1000 FFU of F-MuLV-C were used as positive control. Non-treated bmDCs were utilized as a control for non-specific stimulation of CD8^+^ T cells. Both CD11c- and control-scFv-IDRgag induced strong CD8^+^ T cell activation in contrast to untreated DCs (Fig. 4A.). F-MuLV-C-infected DCs also induced CD8^+^ T cell activation, however to a much lower extent as protein Ag loaded DCs (Fig. 4A). Culturing FV-specific CD8^+^ T cells with bmDCs treated with CD11c-scFv-IDRgag resulted in a higher percentage of activated CD8^+^ T cells compared to the stimulation with bmDCs pulsed with control-scFv-IDRgag (Fig. 4A). Determining the co-expression of the two activation markers CD69 and CD25 on FV-specific CD8^+^ T cells we again detected significantly increased CD8^+^ T cell activation in co-cultures containing DCs loaded with CD11c-scFv-IDRgag compared to control-scFv-IDRgag (Fig. 4B). Of note, activation of FV TCRtg CD8^+^ T cells was antigen-specific, since neither C-opsonized F-MuLV nor scFv-IDRgag loaded DCs were able to activate OVA-specific OT-1 CD8^+^ T cells in co-cultures (data not shown).

Augmented activation of specific CD8^+^ T cells by targeting Ags to CD11c was confirmed using the OVA system. Co-culture of OVA-specific OT-1 CD8^+^ T cells with DCs loaded with 10 µg/ml of CD11c-scFv-IDR_OVA_ resulted in a higher magnitude of CD8^+^ T cell activation relative to the activation of FV-specific CD8^+^ T cells induced by the same amount of CD11c-scFv-IDRgag (Fig. 4C). At lower concentrations, a significant enhancement of CD8^+^ T cell activation was detected by bmDCs treated with CD11c-scFv-IDR_OVA_ compared to control-scFv-IDR_OVA_, which disappeared when the concentration reached 10 µg/ml (Fig. 4C). Since targeting Ags to CD11c was also reported to activate specific CD4 responses [11], we next tested targeting of both FV- and OVA-derived Ags to CD11c for activation of specific CD4^+^ T cells. Compared to control-scFv, both CD11c-scFv-IDRenv and CD11c-scFv-IDR_OVA_ loaded DCs were able to significantly enhance activation of CD4^+^ T cells in co-culture experiments, however differences between CD11c targeted and non-targeted Ags were less prominent as seen with CD8^+^ T cells (Fig. 4D and 4E).

Further analyzing the activation of CD8^+^ T cells, we found that activation induced by DCs loaded with 10 µg/ml of CD11c-scFv-IDRgag was equivalent with DCs loaded with a 20 times molar excess of non-targeted FV matrix protein p15 (MA) [29] extended with the same immunodominant CCLCLTVFL FVgag epitope (Fig. 5A). To prove that the CD11c-scFv-IDRgag acts via CD11c we tested the receptor-specificity of IDRgag targeting by incubating DCs with CD11c-scFv-IDRgag either in the presence of the parental anti-CD11c mAb clone N418 or control mAb. Whereas the anti-CD11c parental mAb was able to significantly reduce CD11c-scFv-IDRgag induced CD8^+^ T cell activation in co-culture experiments, no decrease of CD8^+^ T cell activation was observed using the isotype control Ab (Armenian hamster IgG) or the control parental mAb (anti-human β-gal mAb) (Fig. 5B). Since in our co-culture experiments the ratio of DC:T cells at 1∶1 did not represent a physiological ratio, we performed experiments with different DC:T cell ratios by lowering DC numbers in co-cultures. As shown in Figure 5C decreasing DC number in co-cultures resulted in a reduction in CD8^+^ T cell activation, however the difference in CD8^+^ T cell activation between control-scFv-IDRgag versus CD11c-scFv-IDRgag loaded DCs was observable at all investigated DC:T cell ratios.

Recombinant CD11c-scFv-IDRgag and control-scFv-IDRgag constructs have been produced in *E. coli* BL21(DE3) strain and therefore were likely to contain contaminating LPS. Since the amount of LPS contamination in recombinant proteins used in our experiments, might be crucial for the outcome of CD8^+^ T cell activation by DCs, LPS contamination was carefully analyzed. Commercial *Limulus* Amebocyte Lysate (LAL) tests (Lonza) revealed a substantial, however similar LPS-contamination of the fusion proteins corresponding to about 1.20 EU/ml endotoxin in stocks of recombinant proteins used in our experiments (1.23, 1.18 and 1.10 EU/ml for control-scFv-IDRgag, CD11c-scFv-IDRgag and IDRgag-MAp15 respectively), which reveals on about 10 ng/ml LPS contamination in DC cultures. As a possible consequence of LPS-contamination, DCs matured at comparable level in the presence of CD11c-scFv-IDRgag, control-scFv-IDRgag or IDRgag-MAp15 constructs (Fig. 5D). Furthermore, applying additional amount of LPS did not significantly influence the activation of FV-specific CD8^+^ T cells by DCs loaded with either CD11c-scFv-IDRgag or control-scFv-IDRgag constructs (Fig. 5E).

### Improved Rejection of FBL-3 Tumor Cells in Mice Immunized with CD11c-scFv-IDRgag Loaded DCs

In further experiments we analyzed the effect of targeting IDRgag to bmDCs on activation of CD8^+^ in FV-specific TCRtg mice *in vivo*. Whereas mice injected with CD11c-scFv-IDRgag-treated bmDCs showed a significant higher level of CD8^+^ T cell activation as measured by the percentage of CD69 expressing cells, no significant increase was detected in mice receiving DCs with control-scFv-IDRgag compared to mice injected with non-loaded DCs (Fig. 6A). Next we studied the effect of CD8^+^ T cell activation by scFv-gagIDR *in vivo* on the growth of FV-induced erythroleukemia cell-line FBL-3 bearing the FV gag-encoded cytotoxic T cell specific epitope gPr80^gag^85–93 [25]. When inoculated i.d. into B6 mice FBL-3 cells grow to palpable tumors before they regress due to the induction of FV-specific CD8^+^ T cell responses [25]. To study the potential of DC targeting on the killing of FV-related target cells *in vivo*, wild type B6 mice received bmDCs loaded either with CD11c-scFv-IDRgag or control-scFv-IDRgag prior to tumor challenge. Control mice were injected with non-loaded bmDCs. 24 hours later mice were injected i.v. with FV-specific TCRtg CD8^+^ T cells. FBL-3 cells were inoculated i.d. 48 h after bmDCs injection. Tumor development was monitored daily for the next 9 days by measuring the tumor size. Animals injected with untreated DCs rejected the tumor within 11 days (data not shown) with a maximum tumor size of 3.81±0.61 mm at day 6 (Fig. 6B). We observed a significant difference in size of the tumors between the groups of animals receiving DCs loaded either with control-scFv-IDRgag or CD11c-scFV-IDRgag with a maximum tumor size of 2.85±0.54 mm versus 1.00±0.41 mm at day 5, respectively (Fig. 6B).

### Improved Protection After Vaccination with CD11c-scFv-IDRgag Treated DCs Against FV Challenge

Next we investigated whether DC loaded with CD11c-scFv-IDRgag were capable of inducing protective immunity against retroviruses. When animals were immunized twice with bmDCs loaded with CD11c-scFv-IDRgag or control-scFv-IDRgag in one-week interval and challenged one week after the second immunization, at 4 days post infection (dpi) animals immunized with bmDCs loaded with CD11c-scFv-IDRgag were protected against splenomegaly induced temporarily in B6 animals infected with the high virus dose used in this experiment (Fig. 7A). To determine if vaccination had limited viral replication in the spleen, levels of FV infection were determined in an infectious center assay. At 4 dpi significantly fewer cells were infected in mice that were immunized with CD11c-scFv-IDRgag loaded bmDC compared to the animals that received control-scFv-IDRgag loaded DC (Fig. 7B). Since T-cells were found to be critical for efficient immune responses of mice to Friend retrovirus infection [22], we next analyzed the virus-specific CD8^+^ T cell response in the vaccinated mice. To directly visualize FV-specific CD8^+^ T cells we used the MHC class I tetramer technique. The D^b^-GagL tetramer was specific for the H-2D^b^-restricted, immunodominant gag CTL epitope gPr80^gag^85–93 [25]. At 4 dpi, gating on CD43/CD44 double positive effector CD8^+^ T cells we detected a significantly higher percentage of FV-specific CTLs in mice injected with DCs loaded with CD11c-scFvIDRgag (5.08±0.57% specific CD8^+^ T cells), when compared to mice inoculated with control-scFv-IDRgag loaded DCs (2.08±0.43% specific CD8^+^ T cells) (Fig. 8A and 8B). In contrast, mice injected with non-loaded DC showed nearly no detectable FV-specific CTL responses (Fig. 8A and 8B). Similarly, investigating the number of FV-specific CD8^+^ T cells per 10^7^ CD8^+^ T cells, we found significant higher numbers of FV-specific CTLs in mice vaccinated with bmDC/CD11c-scFv-IDRgag (11803±1855) compared to both mice inoculated with non-loaded or control-scFv-IDRgag loaded DCs (4020±599 and 5782±916, respectively) (Fig. 8C).

## Discussion

Here we demonstrate for the first time the potential of targeting viral proteins to CD11c on DCs for the induction of retrovirus-specific CTL responses. Our recombinant CD11c-scFv-IDRgag fusion protein preferentially bound to DCs and provided significantly improved activation of FV-specific CD8^+^ T cells compared to non-targeted Ags both *in vitro* and *in vivo*. CTLs activated by CD11c-scFv-IDRgag provided better rejection of FV-related tumor cells *in vivo*. Furthermore, we observed an improved protection after vaccination with CD11c-scFv-IDRgag treated DCs against FV challenge.

DCs express several molecules like DEC-205, mannose R, FcRs and CRs to capture Ags and direct them to intracellular compartments essential for effective processing and subsequent priming of T cell activation [3], [4], [5], [6]. Therefore, receptors on DCs capturing Ags are of interest for Ag targeting to improve specific immune responses. In mice, CD11c, the α-subunit of CR4, is preferentially expressed on myeloid DCs [31] allowing us to determine the potential of CD11c targeting on DCs for induction of specific immune responses. Similar to other studies, CD11c-specific scFv constructs bound to bmDCs and selectively targeted CD11c expressing CD11b^high^MCH class II^high^ spleen DCs [19]. Of note, a recently indentified B cell sub-population in mice was characterized by low expression levels of CD11c [32]. In humans, DCs as well as other cells like monocytes/macrophages co-express both CD11b and CD11c on their surface [33]. Since DCs as well as B cells and monocytes are professional antigen presenting cells, the possible influence of these cells on the induction of specific immune responses upon targeting Ags to CD11c should be taken into account. Although, CD11c might not target Ags exclusively to DCs, the constitutively high expression of CD11c on DCs is expected to support preferential delivery of Ags to DCs.

Delivering Ags to CD11c on DCs has been shown to induce specific immune responses merely in tumor models but so far little is known about the potential of targeting Ags to CD11c for the induction of specific CD8 T cell responses in viral infections [19], [20]. Here we used the well established FV model to study the activation of virus-specific CTLs. Similar to tumor immunity, induction of strong anti-viral CTL responses is essential for the control of acute FV infections [34]. Targeting the IDR of FVgag to CD11c on DCs provided significantly improved activation of FV-specific CD8^+^ T cells both *in vitro* and *in vivo*. Expression of CD69 and CD25 on CD8^+^ T cells together with the induction of their proliferation in co-culture experiments with DCs pulsed with CD11c-scFv-IDRgag revealed an efficient cross-presentation of viral peptides by DCs. Compared to non-targeted FV MA p15 extended with the IDRgag (IDRgag-MAp15), 20 times less CD11c-scFv-IDRgag is needed to achieve the same activation of FV-specific CD8^+^ T cells proving the high efficiency of Ag targeting.

CD11c-scFv-IDRgag provided a significantly improved T cell activation when compared to the control-scFv-IDRgag construct, however the effect was not as impressive as with MA p15, which might be explainable by the observed slight binding of the control-scFv-IDRgag to bmDCs. Comparing the strength of induced CD8^+^ T cell activation between FV IDRgag and IDR_OVA_ targeted to CD11c revealed the need of about 10 times less of IDR_OVA_ to induce similar CD8^+^ T cell activation as by CD11c targeted with FV IDRgag. These results imply that both the type of Ags and the functional properties of activated Ag-specific CD8^+^ T cells might be critical for the induction of specific CTL responses. Furthermore, OVA-specific CD8^+^ T cell activation was similar between CD11c-scFv-IDR_OVA_ and control-scFv-IDR_OVA_ at higher concentration suggesting that CD11c targeting has relevance for cross-presentation at suboptimal protein concentration. Since cross-presentation of non-targeted proteins by DCs was shown to be effective at high concentration, CD11c targeting might at least reduce the amount of Ag required for an effective vaccination.

Similarly to other studies, targeting Ags to CD11c on DCs delivered Ags into both MHC class I and MHC class II pathways since beside triggering a CD8^+^ T cell response we could also observe a significant enhancement in activation of both FV- and OVA-specific CD4^+^ T cells (Fig. 3D and 3E.) [11]. The difference of CD4^+^ T cell activation levels between FV-specific and OVA-specific CD4^+^ T cells might be due to the fact that FV-specific CD4 TCR tg animals express the transgenic TCR only on a small portion (about a few percent) of CD4^+^ T cells. Although, in contrast to CD8^+^ T cell activation, targeting Ags to CD11c may be less relevant for the improvement of specific CD4^+^ T cell response, further studies are necessary to investigate the effect of Ag delivery to CD11c for the induction of CD4^+^ T cells especially regarding their help for the generation of virus-specific Abs by B cells.

DC-based vaccines utilizing protein Ags either by *ex vivo* loading of DCs or by *in vivo* delivery to DCs represent promising tool for the induction of specific T cell responses, but require adjuvants to induce protective immunity [35]. Several studies revealed the necessity of maturation signals for DC-mediated induction for strong helper and killer T cell immunity, whereas immature antigen-pulsed DCs were demonstrated to induce tolerance [36]. Microbial ligands, particularly Toll-like receptor (TLR) ligands have been reported to provide sufficient activation of DCs subsequently resulting in a strong induction of effector T cell responses. Especially, TLR-3 ligands poly IC and poly ICLC offered promising adjuvant effects for specific T cell responses induced by DC-targeted protein vaccines [37], [38], [39]. CD11c-scFv-IDRgag and control-scFv-IDRgag constructs used in this study were generated in a bacterial expression system, therefore substantial, but similar amount of contaminating LPS was present in all samples. In line with this, DCs pulsed with CD11c-scFv-IDRgag or control-scFv-IDRgag constructs showed similar mature phenotype as measured by the expression of the co-stimulatory molecule CD86. Since under standard laboratory condition it is difficult to produce LPS free recombinant proteins in bacterial expression systems or to completely remove LPS, we decide to use LPS-containing samples in our experiments considering LPS as an activator of DC maturation. However, we could exclude differential activation of specific T cells due to different amount of LPS contamination, since a) additional amount of LPS did not influence either CD11c-scFv-IDRgag or control-scFv-IDRgag induced T cell activation (Fig. 4D); b) enhanced activation of T cells by DCs loaded with CD11c-scFv-IDRgag was diminished if DCs were pulsed in the presence of the parental anti-CD11c mAb clone N418 (Fig. 4B). Using LPS free proteins the necessity of adjuvants upon CD11c targeted protein vaccination should be further analyzed both *in vitro* and *in vivo*, since the efficacy of targeting protein vaccines to receptors on DCs was dependent on the type of adjuvants used [38], [40].

In FV-specific TCRtg mice CD8^+^ T cells were activated upon injection with bmDCs pulsed with CD11c-scFv-IDRgag to demonstrate the capacity of DCs for virus-specific CD8^+^ T cell activation. Growth of the FV-induced erythroleukemia cell-line FBL-3 has been reported to be controlled by CD8^+^ T cells in about 10 days after inoculation in B6 mice [34]. Using this experimental model we tested whether DCs targeted with CD11c-scFv-IDRgag can better control the growth of FV-related FBL-3 target cells. Compared to control-scFv-IDRgag, targeting FV IDRgag to CD11c resulted in a significant improvement in tumor rejection. This experiment demonstrated that DCs, which were targeted with FV IDRgag through CD11c were able to activate FV-specific T cells and induce a functional CTL response to eliminate specific target cells *in vivo*.

Antigen-loaded DCs as vectors for vaccination against viral infections have been demonstrated to induce protective antiviral immunity [41]. Furthermore, a previous study on DCs loaded with lysate of FV demonstrated the potency of DC vaccine in retroviral infections by inducing specific cellular immune response [42]. In our study we also investigated whether targeting of retroviral antigens to CD11c on DCs is capable of improving DC vaccine induced retrovirus-specific CTL response. Vaccinating mice twice with DCs treated with CD11c-scFv-IDRgag resulted in protection against FV-induced splenomegaly and significantly reduced viremia in challenged animals. Furthermore, as early as 4 dpi we could already detect FV-specific CD8^+^ effector T cells in the vaccinated mice. In line with this observation, induction of virus-specific CTLs upon vaccination of mice with DCs loaded with FV lysate has been demonstrated to be crucial for the protective effect of the DC vaccine [42].

The induction of strong virus-specific T cell responses is critical to control acute viral infections. Previous studies evaluating the efficacy of live attenuated virus vaccination against FV infection revealed an excellent protection requiring the interplay of both CD4+ and CD8+ T cells and also B cells [43], [44]. Nevertheless, safety concerns of live attenuated retroviruses may exclude their use in humans as vaccines. As alternatives, vaccination against retroviral infections either with adenoviral vector encoding retroviral Ags or with envelope related peptides of CD4+ T cell epitopes improved virus-specific CD4+ T cell and neutralizing Ab responses [45], [46]. DC-based therapies vaccinating either with Ag-loaded DCs or by targeting Ags to DCs using DC-specific surface molecules might also represent suitable approaches for the induction of virus-specific immune responses [47]. Beside CD11c a variety of receptors like DEC-205, Clec9A, LOX1, mannose receptor, CD36 and DC-SIGN have been studied for their capacity to deliver Ags to DCs [48], [49], [50], [51], [52], [53]. Protective immunity elicited by DC-targeted vaccines was correlated with the induction of strong CD8+ and CD4+ T cell responses. Activation of DCs represents one of the challenges to establish a successful DC vaccine, since DCs in the absence of inflammatory stimuli or infection are rather tolerogenic [47]. In last years great efforts have been made to find different ways to formulate the adjuvant and vaccine to be most effective for DC vaccination [35], [47]. Here we present first evidence for the improvement of DC vaccine induced retrovirus-specific CD8^+^ and CD4^+^ T cell response by targeting viral Ags to CD11c on DCs both *in vitro* and *in vivo*. Thus, CD11c targeted protein vaccines triggering cellular immunity might provide alternatives to other vaccination strategies or enhance the effectiveness of current strategies. Further investigations have to be focused on protein vaccinations targeting viral Ags to CD11c followed by virus-challenge *in vivo* to elucidate the therapeutical potential of induced anti-viral immune response.
